# *Cantharellus guyanensis* (Basidiomycota, Hydnaceae): A Gourmet Mushroom in the Brazilian Cerrado

**DOI:** 10.1007/s00284-026-04899-x

**Published:** 2026-04-25

**Authors:** Robson Bernardo Silveira-Silva, Felipe Wartchow, Lucas Leonardo-Silva, Fábio Neves Vieira, Solange Xavier-Santos

**Affiliations:** 1 Laboratório de Micologia Básica, Aplicada e Divulgação Científica – FungiLab, Universidade Estadual de Goiás, Anápolis, GO Brazil; 2 Departamento de Sistemática e Ecologia, Universidade Federal da Paraíba, João Pessoa, PB Brazil

## Abstract

*Cantharellus* is an important genus of edible fungi with a worldwide distribution, whose species are recognized for their exceptional taste, beauty, and unique texture. Species of *Cantharellus* are terrestrial ectomycorrhizal fungi valued for their nutritional and medicinal properties, with demonstrated therapeutic potential due to their anti-inflammatory, antimicrobial, and antigenotoxic activities. *Cantharellus guyanensis*, originally described in 1854 from French Guiana, grows on soil and is currently known from restricted areas in South America, including the Amazon and Atlantic forests in Brazil. Appreciated in gourmet cuisine, *C. guyanensis* is reported here for the first time in the Cerrado biome. The species was confirmed through morphological and phylogenetic analysis (ITS, LSU and TEF1-α genetic markers), and the voucher is deposited in the fungaria of the Universidade Estadual de Goiás (HUEG-Fungi) and Universidade Federal da Paraíba (JPB). Its nutritional potential determined by bromatological analysis revealed good storage stability, maintaining its high concentration of general digestible nutrients and low lipid contents. This record expands knowledge about the distribution of the genus among Brazilian biomes and reveals an excellent alternative food resource for the Cerrado.

## Introduction

*Cantharellus* Adans. ex Fr. is the type genus of the order Cantharellales and comprises around 300 species [1]. Regarding its systematics, insights were presented during past years and recognition of at least six subgenera: *Afrocantharellus* Eyssart. & Buyck, *Cantharellus*, *Cinnabarinus* Buyck & V. Hofst., *Parvocantharellus* Eyssart. & Buyck, *Pseudocantharellus* Eyssart. &Buyck and *Rubrinus* Eyssart. & Buyck [1].

For a long time, only *C. guyanensis* was reported from Brazil in the Amazon region [2]. In 1994, Petersen & Mueller described *C. xanthoscyphus* R.H. Petersen from a dense ombrophilous upper-montane forest, and Meijer [3] recorded *C. guyanensis* in an *Araucaria angustifolia* (Bertol.) Kuntze forest. Studies on this genus was restarted by Wartchow et al. [4, 5], Pinheiro & Wartchow [6], Henkel et al. [7] and Nascimento et al. [8], who described or cited species such as *C. aurantioconspicuus* Wartchow & Buyck, *C. amazonensis* Wartchow, *C. protectus* Wartchow & Pinheiro, *C. guyanensis* and *C. rubescens* Nascimento, Pinheiro, Wartchow & Alves in the Atlantic Forest and Caatinga biomes, in the Northeast of Brazil.

Species of *Cantharellus*, including *C. guyanensis* [9], are terrestrial ectomycorrhizal fungi [1] known for their nutritional and medicinal value [10]. They are used in European and Asian folk medicine to treat liver, lung and stomach disease. Several studies highlight their anti-inflammatory, antimicrobial and antigenotoxic action, reinforcing their therapeutic potential, expanding the possibilities for medicinal application and their relevance in the health field [10–13].

These mushrooms are known for their content of vitamin A, derived from carotenoids, and vitamin D, both essential for human health [14–16]. In addition, *Cantharellus* are among the most valued wild edible fungi in the world. Recognized globally, they stand out not only for their exceptional taste, but also for their distinctive beauty and unique texture [17, 18].

Species of *Cantharellus* are widely distributed and are especially rich in subtropical to tropical zones [1, 19, 20]. Their vibrant color, ranging from shades of orange and yellow, combined with a fruity aroma reminiscent of apricots, makes them a highly coveted ingredient in cuisine [21]. This distinctive aroma, along with their refined flavor, appealing color, and unique texture, has made them legendarily known in European woodlands for the sweet, apricot-like scent they exude [17, 22].

Their reputation dates back to the 19th century, when they were described and named by Fries in 1821 [23], who declared them to be one of the most important and best edible fungi. In European gastronomy, they are often used in sophisticated preparations. This combination of unique characteristics consolidates *Cantharellus* as one of the most desired fungi both in the kitchen and in nature [21]. Its popularity as a natural ingredient in medicinal and gastronomic practices highlights the importance of studies exploring its nutraceutical properties. This study aims to report, for the first time, the collection of *C. guyanensis* in the Cerrado biome, characterizing it morphologically and phylogenetically, as well as its nutritional potential.

## Materials and Methods

### Study Area

*Cantharellus* basidiomata were collected at “*Serrinha do Paranoá*” (15°43′02″ S 47°49′45″ W), a Cerrado fragment of approximately 3026 ha and 1100 m altitude [24], located northeast of Lake Paranoá, in Brasília, Distrito Federal, Brazil. The predominant vegetation in the area is “*cerrado rupestre*”, characterized by shrubby-woody or sub-shrubby-herbaceous formations on slopes, with sandy soil in crevices between rocks [25].

### DNA Extraction, Amplification, and Sequencing

Species identification was based on phylogenetic and morphological analyses. For the phylogenetic analyses, total genomic DNA was extracted from fragments of fresh basidioma, macerated in liquid nitrogen, according to the CTAB method [26]. The extracted DNA was quantified with a spectrophotometer using Qubit (Invitrogen). The ITS, LSU and TEF1-α regions were then amplified with the primers ITS5/ITS4 [27], LR0R/LR5 [28] and EF1-983 F/EF1-1567R [29], respectively. The amplification products were purified and sequenced with the same primers used in the amplification, on an Applied Biosystems 3730xl DNA sequencer (MacroGen Ltd., South Korea).

### Phylogenetic Analyses

The DNA sequences obtained in this study were assembled and edited using Staden Package v. 2.0 [30]. The consensus sequence was aligned with sequences from the Hydnaceae family (Table 1) by searching the BLAST database (http://blast.ncbi.nlm.nih.gov/) using the online version of MAFFT [31] in “Auto” mode for strategy, and then manually edited in MEGA v.6 [32]. *Clavulina cristata* (Holmsk.) J. Schröt. was used as an outgroup in the phylogenetic analyses, according to [33]. Each genetic marker (ITS, LSU and TEF1-α) was aligned individually before assembling the combined dataset.

**Table 1:** Sequences available on GenBank and used in phylogenetic analysis. The sequence obtained in this study is marked in bold. T = type; I = isotype

Species	Voucher	Origin	ITS	LSU	TEF1-α	Reference
*Cantharellus albidolutescens*	BB 08.057	Madagascar	-	KF294645	KF294752	Buyck et al. [19]
*Cantharellus albidolutescens* **T**	BB 08.070	Madagascar	KF981365	KF294646	JX192982	Buyck et al. [19]
*Cantharellus ambohitantelyensis* **T**	BB 08.336	Madagascar	KF981366	KF294656	JX192989	Buyck et al. [19]
*Cantharellus cibarius*	GE 07.025	France	KX907204	KF294658	GQ914949	Olariaga et al. [40], Buyck et al. [19, 41]
*Cantharellus cibarius* **T**	BIO-Fungi 10,986	Sweden	KR677501	KR677539	KX828823	Olariaga et al. [40, 42]
*Cantharellus cinnabarinus*	BB 07.053	USA	-	KF294630	GQ914984	Buyck et al. [19, 41]
*Cantharellus cinnabarinus* **T**	BB 07.001	USA	-	KF294624	GQ914985	Buyck et al. [19, 41]
*Cantharellus coccolabae*	1064/RC 14.24	Guadeloupe	-	KX857088	KX857020	Buyck et al. [20]
*Cantharellus coccolabae*	1065/RC 11.25	Guadeloupe	-	KX857089	KX857021	Buyck et al. [20]
*Cantharellus decolorans*	BB 08.278	Madagascar	KX907203	KF294654	GQ914968	Olariaga et al. [40], Buyck et al. [19, 41]
*Cantharellus garnieri*	1020/BB 09.024	New Caledonia	-	KX857085	KX857017	Buyck et al. [20]
*Cantharellus garnieri*	1021/BB 09.033	New Caledonia	-	KX857086	KX857018	Buyck et al. [20]
***Cantharellus guyanensis***	**HUEG17234**	**Brazil**	**PV845098**	**PV844828**	**PV930050**	**Present study**
*Cantharellus guyanensis*	1517/MR	Guyana	-	KX857095	KX857061	Buyck et al. [20]
*Cantharellus guyanensis*	1501/MRG07	Guyana	-	KX857094	KX857060	Buyck et al. [20]
*Cantharellus guyanensis*	TH9732	French Guiana	KC897654	KC897656	-	Henkel et al. [7]
*Cantharellu sguyanensis*	THV18	Venezuela	-	KC897657	-	Henkel et al. [7]
*Cantharellus guyanensis*	JPB46806	Brazil	-	KC897659	-	Henkel et al. [7]
*Clavulina cristata*	JKU8	USA	JN228227	JN228227	-	Uehling et al. [44]
*Craterellus atratoides*	TH8473	Guyana	JQ915103	JQ915129	-	Wilson et al. [45]
*Craterellus atratoides* **T**	TH9232	Guyana	JQ915111	NG042660	-	Wilson et al. [45]
*Craterellus atratus*	MCA1070	Guyana	JQ915092	JQ915118	-	Wilson et al. [45]
*Craterellus atratus*	MCA990	Guyana	JQ915100	JQ915126	-	Wilson et al. [45]
*Craterellus cornucopioides*	WA0000071019	Poland	MK028881	-	-	Kotowski et al. [46]
*Craterellus cornucopioides*	groc_11399	USA	KT693262	-	-	Raja et al. [47]
*Craterellus cornucopioides*	4525	Italy	JF907967	-	-	Osmundson et al. [48]
*Craterellus excelsus*	MCA3107	Guyana	JQ915095	JQ915121	-	Wilson et al. [45]
*Craterellus excelsus* **I**	TH8235	Guyana	JQ915102	JQ915128	-	Wilson et al. [45]

Phylogenetic reconstructions were inferred by Maximum Likelihood (ML) and Bayesian Inference (BI). Three partitions (ITS, LSU and TEF1-α) were established to estimate the best evolutionary model based on the Akaike Information Criterion (AIC), using ModelTest in TOPALi 2.5. ML analysis was performed in W-IQ-TREE [34], using the GTR + G model, with branch support (BS) inferred by 1000 bootstrap replicates. BI analysis was performed in MrBayes v.3.2 [35] for two independent runs, each with four chains and run for 6 million generations, with convergence verified in the TRACER v. 1.7.1 program [36]. The first 25% of the trees generated were discarded as burn-in, and Bayesian posterior probabilities (PP) were calculated from the remaining trees. The statistical support of the branches was considered strong for BS and UB ≥ 70% and PP ≥ 0.95.

### Morphological Analysis

Microscopic characterization and measurements were made using freehand sections of fresh material, placed in solutions of 3% KOH, Congo red and Melzer’s reagent. Analysis of the basidiospores followed the methodology of Tulloss et al. [37], considering 30 spores measured in side-view. The abbreviations adopted include: L(W), which represents the average length (width) of the basidiospores; Q, which corresponds to the range of the length/width ratio considering all the basidiospores measured; and Qm, which indicates the average Q calculated from all the spores analyzed. For all other microstructures, 10 individuals were measured. The basidioma colors code followed the color chart of Kornerup and Wanscher [38]. The voucher was preserved and deposited in the deposited in the fungaria of the Universidade Estadual de Goiás (HUEG-Fungi) and Universidade Federal da Paraíba (JPB) [39]. Geographic distribution was determined based on localities reported in the literature, and the distribution map was generated using Quantum GIS (QGIS) software [40].

### Nutritional Composition

Approximately 20 g of fresh basidiomata were collected and transported to the laboratory for bromatological and mineral analyses. The material was initially dehydrated in a forced-air oven at 40 °C until constant weight and, after pre-drying, manually ground using a mortar and pestle until a homogeneous powder was obtained.

Moisture and dry matter contents were determined gravimetrically based on the difference between fresh and dry weights. Mineral matter (ash) was obtained by incineration of the dried material in a muffle furnace at 550 °C for approximately four hours. Total nitrogen was quantified using the Kjeldahl method, and crude protein content was estimated using a conversion factor of 6.25. Acid detergent insoluble nitrogen, neutral detergent insoluble nitrogen, non-protein nitrogen, and crude protein solubility in 0.2% KOH were also determined. Ether extract (crude fat), crude fiber, acid detergent fiber (ADF), neutral detergent fiber (NDF), lignin, cellulose, hemicellulose, starch, organic carbon, titratable acidity (NaOH), total digestible nutrients, total carbohydrates, and non-fibrous carbohydrates were evaluated on a dry matter basis. The levels of macroelements (N, P, K, Ca, Mg, and S) and microelements (Mo, Na, Cu, Fe, Mn, Zn, and Co) were also determined.

All determinations strictly followed the official analytical methods of the Brazilian Ministry of Agriculture, Livestock and Food Supply (MAPA) [41], the Brazilian Compendium of Animal Feed [42], and Malavolta et al. [43]. Results were expressed according to the units established by these methods and are presented as mass percentage (% w/w, dry weight basis), g kg⁻¹, or mg kg⁻¹, as appropriate for each constituent.

## Results

### Phylogenetic Analyses

**Fig. 1:**
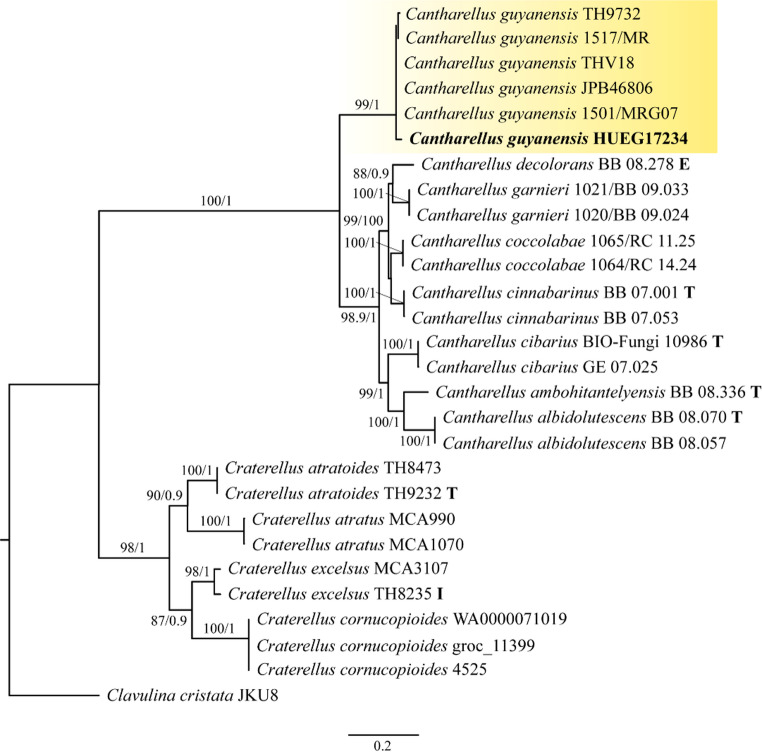
Phylogenetic tree obtained from the ITS + LSU + TEF1-α genetic markers . The topology of the tree is from the maximum likelihood analysis and the sequence generated in this study is indicated in bold. The numbers on the branches indicate the bootstrap frequency (BS) / posterior probability (PP) values

In the phylogenetic analysis, the combined ITS + LSU + TEF1-α dataset included sequences from 28 specimens of the Hydnaceae family, including the outgroup of the family Clavulinaceae. The alignment resulted in a total of 3406 characters, including gaps (ITS: 1-1095; LSU: 1096–2567; TEF1-α: 2568–3406), of which 1800 were constant, 1606 variable and 1322 informative parsimony. The following evolutionary models were used for the analysis: HKY + G for ITS; GTR + G for LSU; and TIMef + G for TEF1-α. The ML and BI analyses produced trees with similar topologies; therefore, only the ML tree is shown (Fig. 1). The *Cantharellus* species recovered in this study formed a clade with a high support value (BS = 100, PP = 1), while *C. guyanensis* emerged as a sister group to the other species in the genus, also with a strong support value (BS = 99, PP = 1).

### Morphological Analysis

**Fig. 2:**
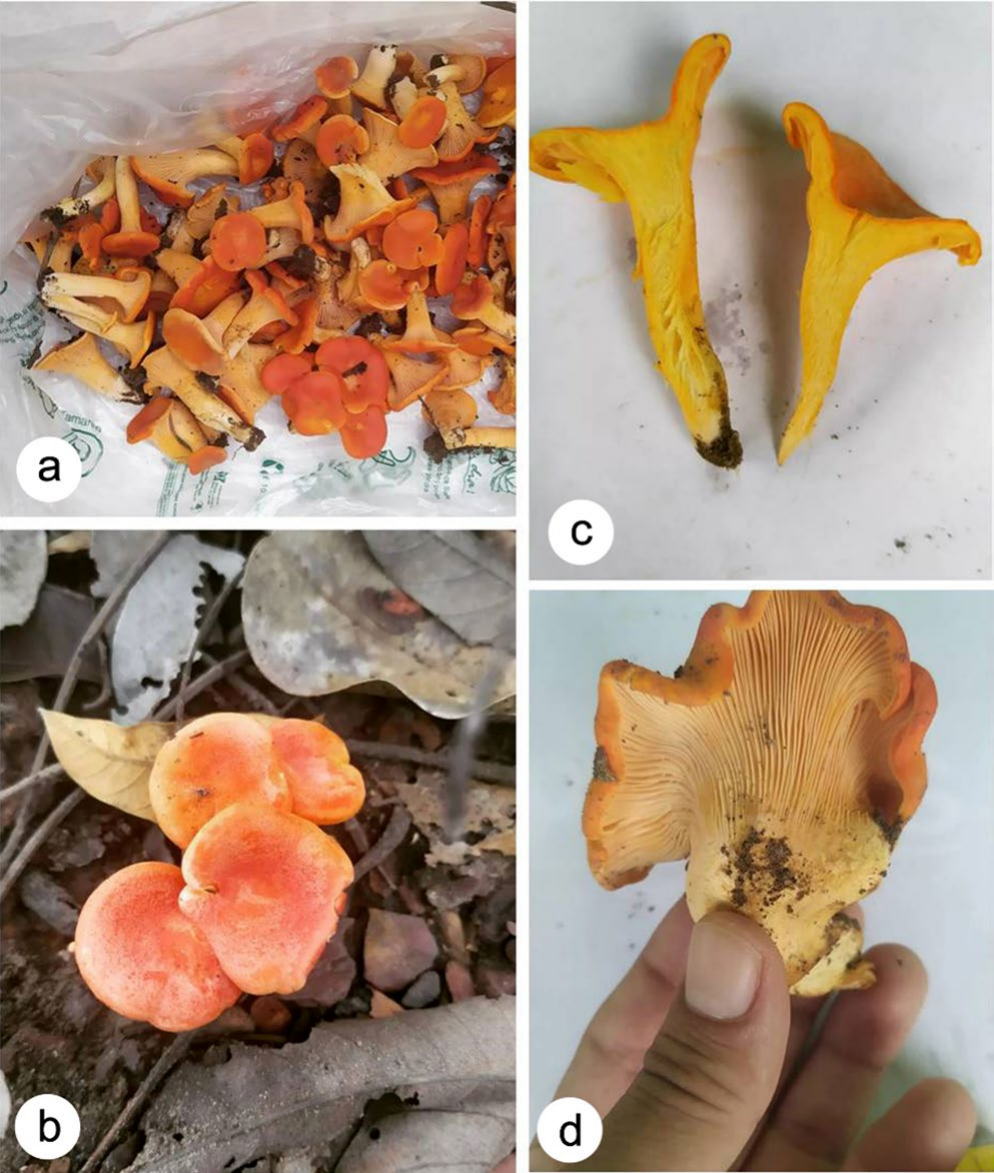
*Cantharellus guyanensis.* **A-B**. Top and side view of the basidiomata. **C**. Context. **D**. Hymenophore

**Fig. 3:**
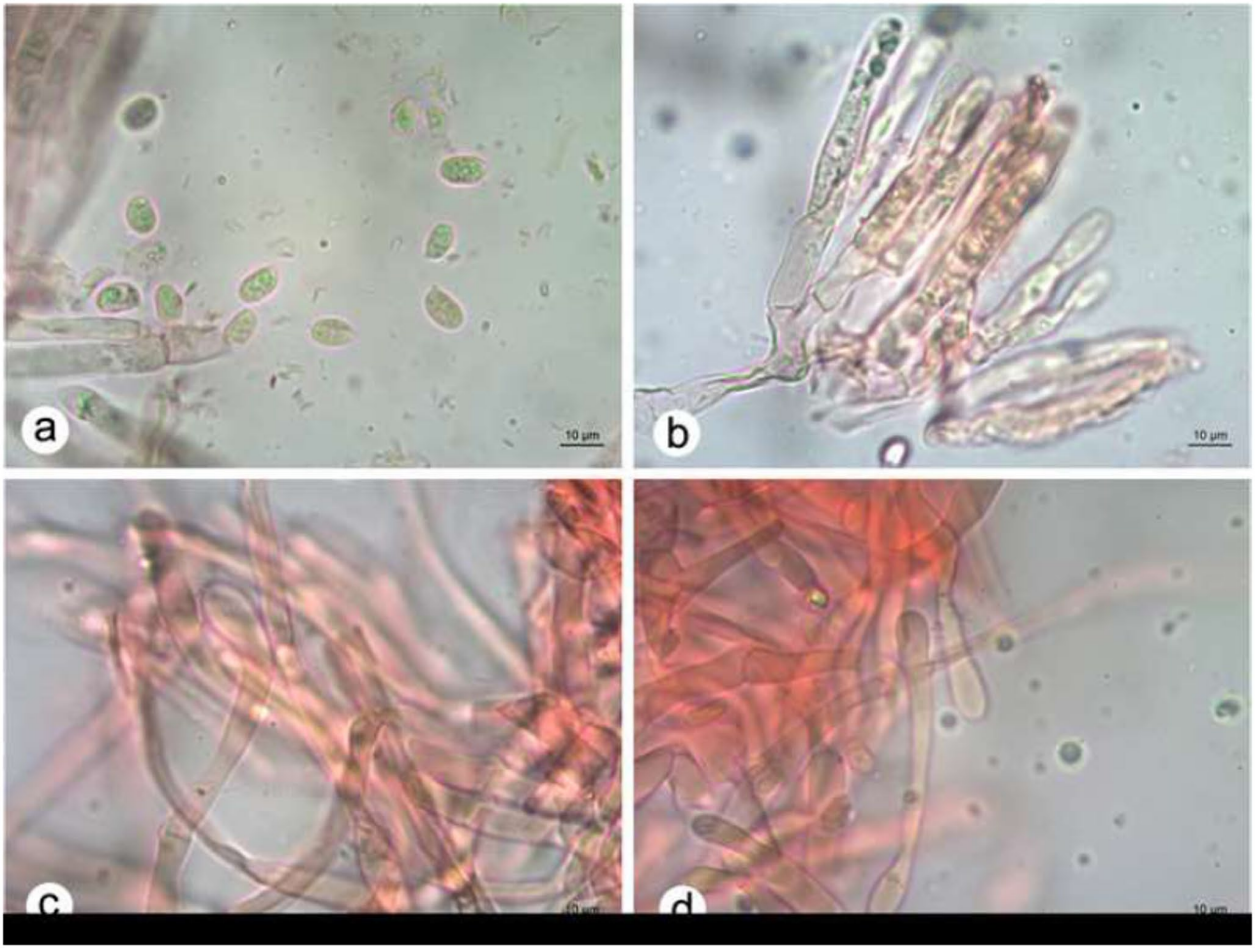
Microstructures of *Cantharellus guyanensis*. **A**. Basidiospores. **B**. Basidia, basidioles and adjacent cells of the subhymenium.**C-D**. Terminal elements of the pileipellis

***Cantharellus guyanensis*** Mont., Annls Sci. Nat., Bot. ser. 4 1: 107 (1854) (Figs. 2 and 3).

Material examined: BRAZIL - Distrito Federal, Brasília, Serrinha do Paranoá, 7°21′50″ S 39°19′45″ W, 31 March 2024, *F.N. Vieira* s/n (HUEG17234; JPB68575).

**Basidiomata** gregarious, small, fleshy. **Pileus** up to 40 mm in diam., mostly concave and widely depressed to shallowly infundibuliform; surface smooth to squamulose-furfuraceous, waxy or dry, slightly shiny, reddish orange (7A8) to yellowish red (8A8), becoming paler in older specimens; margin entire, involute in young basidiomata then incurved to slightly even, smooth, wavy, frequently lobed; context thick at centre; context thick at centre, yellowish orange (4B7), unchanging. **Hymenophore** formed by close to subclose folds, that are dichotomously forked and anastomosing (about 1–1.5 mm broad), decurrent, pale cream to cream then yellowish spots; edge smooth, even and obtuse. **Stipe** up to 16.7 × 5.3 mm, mostly tapering downward, sometimes very stout with somewhat similar width in all length; surface smooth, glabrous, cream to yellowish, unchanging even when handled; context solid, yellowish, unchanging. **Odor** very pleasant and conspicuously fruity when fresh; farinose in dried state; taste astringent but agreeable in fresh specimens. **Spore print** not obtained.

**Basidiospores** (6.8–)7–9(–9.8) × 4.5–5.5(–6) µm, L = 8 μm; W = 5 μm; Q = (1.33–)1.38–1.87 (–1.93); Q = 1.61, inamyloid, hyaline in 3% KOH, ellipsoid to elongate, reniform in side view, smooth, thin-walled, filled with small guttules; hilar appendix prominent, sublateral. **Basidia** 50–60 × 6.5–7.5 μm, slender-clavate, colorless, 4–5–6-spored, clamped. **Basidioles** abundant, subcylindric to nearly narrowly clavate. **Hymenial cystidia** absent. **Subhymenium** containing inflated elements 6.5–9.5 μm wide, from where basidia and basidioles arise, colorless, mostly thin-walled. **Hymenophoral trama** with strongly interwoven hyphae 3–7.5 μm wide, repeatedly branched, colorless, thin-walled. Pileus context consisting of loosely interwoven hyphae. **Pileipellis** composed of some densely packed and prostate hyphae, sometimes periclinally interwoven, mostly periclinal, 3–5.5 μm wide, pale pigmented, thick-walled 0.8–1 μm thick; **terminal elements** 31–56 × 5–6 μm, slender clavate, wall up 0.5 μm thick, sometimes anticlinal. **Clamp connections** abundant and conspicuous in all examined tissues.

#### Habitat

gregarious and scattered, emerging from sandy soil, among some trees of “*cerrado rupestre*”vegetation.

#### Known distribution

**Fig. 4:**
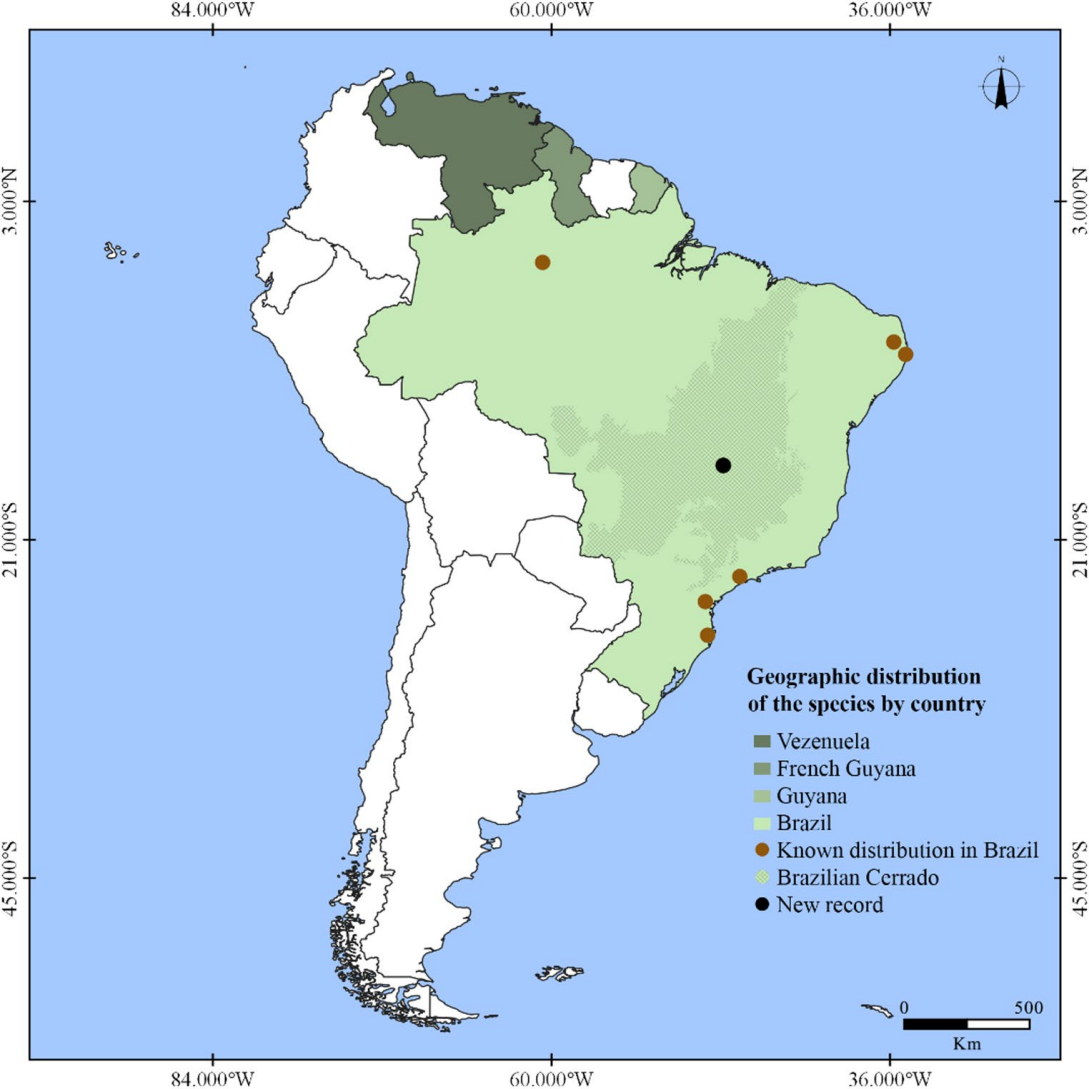
Geographical distribution of *Cantharellus guyanensis*

French Guyana, Guyana, Venezuela [7] and Brazil in the states of Amazonas [2], Distrito Federal (present study), Paraíba, Paraná, Pernambuco [2, 3, 7], Santa Catarina and São Paulo [44] (Fig. 4).

#### Remarks

*Cantharellus guyanensis* has some peculiar features and is recognized in the field by the small orange yellow basidiomata, hymenophore composed of dichotomously forked and sometimes anastomosed folds; the odor is very pleasant and conspicuously fruity when fresh and pleasant farinose in dried state, and the taste astringent but agradable in fresh specimens in our specimens. Microscopically, it bears (1) ellipsoid to cylindrical, smooth, thin-walled, basidiospores (6.8–)7–9(–9.8) × 4.5–5.5(–6) µm, (2) slender-clavate, mostly 4–5–6-spored basidia, (3) and sometimes presenting thick-walled (to 1 μm thick) densely packed hyphae in the pileipellis bearing slender clavate terminal elements.

### Nutritional Composition

*Cantharellus guyanensis* had 96% dry matter and 4% moisture, with 8.8% mineral material. The crude protein content was 25%, with high solubility in 0.2% KOH (85.2%), indicating good protein availability and easy digestibility. Total carbohydrates reached 64.2%, with 42% non-fiber carbohydrates and 42% starch, reinforcing its energy potential. The total digestible nutrient content was 79.8%, while crude fat accounted for just 2%.

The fibrous fraction included 5% crude fiber, 10.6% acid detergent soluble fiber and 22.2% neutral detergent soluble fiber, with lower levels of lignin (6.8%), cellulose (3.8%) and hemicellulose (11.6%). Among the macro and micronutrients, 40 g/kg of nitrogen, 28.8 g/kg of potassium and 347 mg/kg of iron stand out, the latter in high concentration. Elements such as copper (63 mg/kg), manganese (45 mg/kg) and zinc (73 mg/kg) were also quantified, reinforcing the species’ nutritional potential (Table 2).

**Table 2:** Nutritional composition of *Cantharellus guyanensis*

**Nutrient constituent**	Dry matter (%)	96
	Humidity (%)	4
	Mineral matter (%)	8.8
	Crude protein (%)	25
	Crude protein solubility in 0.2% KOH (%)	85.2
	Crude fat (%)	2
	Crude fiber (%)	5
	Acid detergente soluble fibers (%)	10.6
	Neutral detergente soluble fibers (%)	22.2
	Acid detergent in soluble nitrogen (%)	3.9
	Neutral detergente insoluble nitrogen (%)	1.4
	Non-protein nitrogen (%)	0.1
	Total carbohydrates (%)	64.2
	Non-fibrous carbohydrates (%)	42
	Total digestible nutrients (%)	79.8
	Acidity (NaOH) (mg NaOH)	2.3
	Organic carbon (%)	52.7
	Lignin (%)	6.8
	Starch (%)	42
	Cellulose analysis (%)	3.8
	Hemicellulose (%)	11.6
**Macroelements**	N (g/kg)	40
	P (g/kg)	3.6
	K (g/kg)	28.8
	Ca (g/kg)	0.9
	Mg (g/kg)	1.3
	S (g/kg)	1.4
**Microelements**	Mo (mg/kg)	2
	Na (g/kg)	0.5
	Cu (mg/kg)	63
	Fe (mg/kg)	347
	Mn (mg/kg)	45
	Zn (mg/kg)	73
	Co (mg/kg)	4

## Discussion

*Cantharellus guyanensis* was originally described from French Guiana in 1854, occurring on soil [49]. To date, its known distribution has been restricted to a few South American countries, with records in Brazil limited to the Amazon and Atlantic forests [2, 3], and the Guiana Shield [7]. The species is an ectomycorrhizal fungus [9], and the discovery of the species in the Cerrado led us to discuss on the ecological aspects of the site collection (i.e., “*cerrado rupestre*”) and the putative host tree.

The collection site corresponds to a “*cerrado rupestre*” formation, characterized by sloping terrain, shallow sandy soils with low nutrient availability, and a predominantly shrubby–herbaceous vegetation layer ranging from 2 to 4 m in height [25]. Floristic surveys of the area recorded 51 plant species across 25 families, including representatives of Fabaceae, Melastomataceae, Malpighiaceae, Vochysiaceae, Asteraceae, Bignoniaceae, and Rubiaceae [45]. The survey also documented the occurrence of *Guapira gracilliflora* (Schmidt) Lundell and *G. noxia* (Netto) Lundell (Nyctaginaceae), tree species belonging to a genus recently confirmed to form ectomycorrhizal associations based on anatomical root studies [9]. In that study, 10 distinct ectomycorrhizal morphotypes were identified on the roots of *G. opposita* (Vell.) Reitz in a “*restinga*” forest in southern Brazil. Notably, this vegetation type shares environmental characteristics with the “*cerrado rupestre*”, particularly sandy soils and a predominantly shrubby vegetation structure, suggesting similar ecological conditions for the establishment of ectomycorrhizal fungi [46].

Morphologically, *C. guyanensis* can be distinguished from other Brazilian representatives of the genus. *Cantharellus aurantioconspicuus*, found in the Atlantic Forest, is distinguished by the thin-walled hyphal ends on the surface of the pileus [5]. *Cantharellus protectus*, also from the Atlantic Forest (Paraíba), is similar in its yellow-orange coloration, but differs in having a basidioma that fades to yellow, shorter basidiospores (5.5‒7.5(‒8) × (3‒) 3.5‒5(‒5.5) µm) and a non-fruity odor [6]. *Cantharellus amazonensis*, from the Central Amazon, is distinguished by its bright red pileus, non-anastomosing lamellae and thin-walled pileipellis terminal elements [4]. On the other hand, *C. rubescens*, from the Caatinga (Ceará), has a rubescent basidiomata, a scaly stipe and significantly wider pileipellis terminal elements (20‒64 × 6–13 μm) [8].

Nutritional composition indicates that *C. guyanensis* has a nutritional profile with a high concentration of nutrients and good storage stability, due to its high dry matter content and low moisture content. The crude protein content and its high solubility reinforce its potential as a viable protein source, with good digestibility and bioavailability of amino acids. This characteristic brings it closer to widely marketed mushroom species, suggesting that it may have relevance as a comparable food [47]. From a comparative perspective, this protein content (25%) falls within the range reported for the well-known edible *C. cibarius* (20–27%) [17] and is similar to values described for cultivated mushrooms such as *Pleurotus ostreatus* (Jacq.) P. Kumm. (21,5%) [48], which are recognized for their moderate-to-high nutritional quality. Moreover, the protein concentration observed here exceeds those reported for several native Cerrado macrofungi, including *Auricularia nigricans* (Sw.) Birkebak, Looney & Sánchez-García (7%), *Schizophyllum commune* Fr. (10%), and *Lentinus crinitus* (L.) Fr. (14%), and approaches the levels described for *Favolus brasiliensis* (Fr.) Fr. (27%), another nutritionally relevant regional species [49, 50].

The predominance of total carbohydrates, particularly non-fibrous fractions, combined with a high proportion of total digestible nutrients, highlights its energetic potential. In parallel, the relatively low lipid fraction (2%) follows the general pattern of edible mushrooms, which are typically characterized by minimal fat content and favorable digestibility, making the species suitable for low-lipid, high-energy diets. Compared with more structurally fibrous Cerrado taxa, such as *F. brasiliensis* and *L. crinitus*, which present fiber levels between 17 and 26%, the lower fiber content observed in *C. guyanensis* may further enhance palatability and digestibility. The mineral composition, particularly the elevated concentrations of iron, potassium, zinc, copper, and manganese, together with low sodium levels, reinforces its nutritional relevance, as edible mushrooms are increasingly recognized as important dietary sources of essential micronutrients. These compositional attributes, combined with its pleasant odor and taste and its frequent occurrence in the collection area, support its potential acceptance as a regional food resource.

Beyond its proximate composition, the pleasant aroma and taste observed in the field likely contribute to its culinary appeal. Species of *Cantharellus* are known to produce characteristic volatile organic compounds, especially C8 derivatives such as 1-octen-3-ol and 3-octanone, as well as terpenoid and apocarotenoid compounds responsible for the fruity and mushroom-like notes typical of chanterelles [51, 52]. Although volatile profiles were not analyzed here, the sensory properties reported are consistent with these chemical patterns.

Overall, these attributes indicate that *C. guyanensis* presents a compositional profile comparable to nutritionally valuable edible fungi. However, it is important to acknowledge methodological limitations: bromatological analyses were conducted on a single pooled sample due to limited basidioma availability, preventing replicate measurements and the estimation of variability parameters. Consequently, the results should be interpreted as descriptive rather than population-level estimates.

Despite this limitation, the potential edibility of *C. guyanensis* is supported not only by its favorable nutritional composition but also by the broader context of wild mushroom consumption in Brazil. A recent Brazilian survey documented more than 400 wild edible mushroom species in the country, including 350 taxa considered safe for consumption, underscoring the considerable yet underutilized diversity of native fungi as food resources [44]. Ethnomycological, taxonomic, and molecular evidence supports the evaluation of native fungal species within the framework of food security and the sustainable use of biodiversity. In this context, *C. guyanensis* stands out as a promising edible macrofungus from the Brazilian Cerrado, combining favorable nutritional characteristics, including high protein content, low lipid levels, palatability, and local availability. Its occurrence in the Cerrado also extends the known geographical distribution of the species beyond the Amazon and Atlantic forests, reinforcing the potential of native mushrooms as alternative food and nutraceutical resources that may contribute to dietary diversification and more sustainable human nutrition.

## Data Availability

Voucher is deposited in the fungaria of the Universidade Estadual de Goiás (HUEG-Fungi) and Universidade Federal da Paraíba (JPB). The sequences generated in this study are deposited in NCBI GenBank (https://www.ncbi.nlm.nih.gov/genbank/).
